# Establishment and Characterization of Mild Atopic Dermatitis in the DNCB-Induced Mouse Model

**DOI:** 10.3390/ijms241512325

**Published:** 2023-08-01

**Authors:** Rebecca Riedl, Annika Kühn, Denise Rietz, Betty Hebecker, Karl-Gunther Glowalla, Lukas K. Peltner, Paul M. Jordan, Oliver Werz, Stefan Lorkowski, Cornelia Wiegand, Maria Wallert

**Affiliations:** 1 Department of Dermatology, University Hospital Jena, Dermatological Research Laboratory, 07747 Jena, Germany; rebecca.riedl@uni-jena.de (R.R.); denise.rietz@med.uni-jena.de (D.R.); c.wiegand@med.uni-jena.de (C.W.); 2 Department of Nutritional Biochemistry and Physiology, Institute of Nutritional Science, Friedrich Schiller University, 07743 Jena, Germany; annika.kuehn@uni-jena.de (A.K.); betty.hebecker@uni-jena.de (B.H.); stefan.lorkowski@uni-jena.de (S.L.); 3 Competence Cluster for Nutrition and Cardiovascular Health (nutriCARD) Halle-Jena-Leipzig, 07743 Jena, Germany; 4 Service Unit Experimental Biomedicine, Friedrich Schiller University, 07745 Jena, Germany; karl-gunther.glowalla@uni-jena.de; 5 Department of Pharmaceutical/Medicinal Chemistry, Institute of Pharmacy, Friedrich Schiller University, 07743 Jena, Germany; lukas.klaus.peltner@uni-jena.de (L.K.P.); paul.jordan@uni-jena.de (P.M.J.); oliver.werz@uni-jena.de (O.W.); 6 Jena Center for Soft Matter (JCSM), Friedrich Schiller University, 07743 Jena, Germany

**Keywords:** DNCB, mild atopic dermatitis, extrinsic, atopic dermatitis mouse model

## Abstract

In dermatological research, 2,4-dinitrochlorbenzene (DNCB)-induced atopic dermatitis (AD) is a standard model as it displays many disease-associated characteristics of human AD. However, the reproducibility of the model is challenging due to the lack of information regarding the methodology and the description of the phenotype and endotype of the mimicked disease. In this study, a DNCB-induced mouse model was established with a detailed procedure description and classification of the AD human-like skin type. The disease was induced with 1% DNCB in the sensitization phase and repeated applications of 0.3% and 0.5% DNCB in the challenging phase which led to a mild phenotype of AD eczema. Pathophysiological changes of the dorsal skin were measured: thickening of the epidermis and dermis, altered skin barrier proteins, increased TH1 and TH2 cytokine expression, a shift in polyunsaturated fatty acids, increased pro-resolving and inflammatory mediator formation, and dysregulated inflammation-associated gene expression. A link to type I allergy reactions was evaluated by increased mast cell infiltration into the skin accompanied by elevated IgE and histamine levels in plasma. As expected for mild AD, no systemic inflammation was observed. In conclusion, this experimental setup demonstrates many features of a mild human-like extrinsic AD in murine skin.

## 1. Introduction

Atopic dermatitis (AD) is a common inflammatory skin disease with a prevalence of 15–20% in children [1] and up to 3% in adults [2]. The main characteristics of AD patients are epidermal defects, acanthosis, spongiosis, inflammation, and dysregulation of immune cell interaction. AD is distinguished by acute or chronic stages with shifts in immune cell profiles [3,4]. Eczematous lesions of acute stages are bright red with serous exudate and oozing lesions while chronic lesions are pink to dull red and characterized by lichenification and scaly plaques [4]. Moreover, AD is associated with type I allergies, such as allergic asthma or food allergies, described as the so-called ‘atopic march’ [5,6]. The extrinsic form of AD is characterized by high levels of serum IgE in contrast to the intrinsic form [7,8,9]. The skin disease comes with several co-morbidities, such as depression or social anxiety, contributing to further impairment of life quality [10,11,12].

Skin disease studies and the expansion of knowledge in the field of novel therapeutic compounds are conducted by employing a variety of animal models. Human-like AD skin lesions for pre-clinical studies can be induced by using (i) mice with spontaneously developing skin eczema, (ii) mice that are genetically modified, or (iii) chemically sensitized mice [13,14]. The latter is the most common. 2,4-Dinitrochlorobenzol (DNCB) is a standard compound for inducing contact sensitization and the formation of multiple haptens with extracellular and intracellular proteins in human skin [15]. Repeated DNCB irritation of the murine skin is described in two phases: a sensitization phase (first contact with the hapten) and a challenging phase (second hapten encounter) [16,17,18,19,20,21,22,23].

However, according to published studies, the reproducibility of the DNCB model is highly challenging. Often, the description of the methodology is lacking in vital information on the handling of mice, scoring parameters, or housing and hygienic conditions. Moreover, studies reported highly variable DNCB concentrations and application volumes and frequencies as well as time regimes. Yet, many authors do not precisely describe the AD phenotypes and endotypes such as acute or chronic stages or extrinsic versus intrinsic AD which are present in their model.

The 2010/63/EU directive on the protection of animals used for scientific purposes stipulates reducing the number of animals in experiments, finding alternative methods, and refining the experimental conditions [24]. A prerequisite to reaching these goals is to give access to a comprehensive and detailed description of the methodology which facilitates reproduction and prevents pre-experiments for the establishment as well as the characterization of the disease and description of the transferability of the models to human diseases.

We, therefore, established the DNCB-induced human-like AD mouse model (hlAD model) from the literature with the aim to develop a re-constructible, affordable, and reliable protocol while assessing a broad spectrum of analytical parameters and providing a detailed characterization of the achieved AD phenotype and endotype with regard to human AD. Our work aims to contribute to a better understanding of the DNCB-induced hlAD model and to help other scientists to make reliable choices for their study design with a focus on testing novel therapeutic compounds.

## 2. Results

### 2.1. Effects of DNCB on the Disease Severity

**Figure 1:**
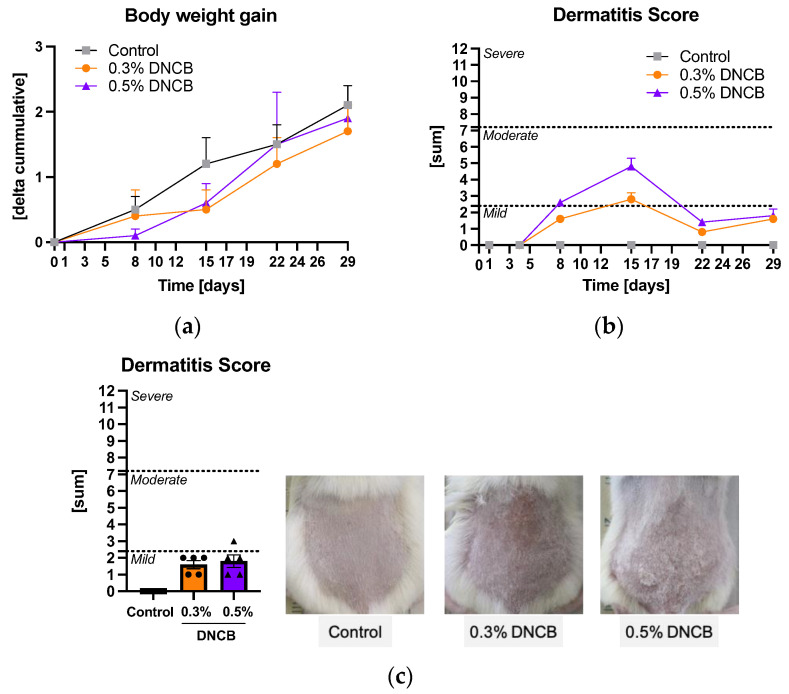
(**a**) Cumulative body weight gain of BALB/c mice during the experiment; (**b**) Dermatitis score of the treated area during the experiment; (**c**) Dermatitis score and images of the treated area at day 29. Data are presented as the mean ± standard error of the mean (SEM) (*n* = 5/group). Squares, dots and triangles represent one individual.

The hlAD was induced by sensibilization of murine skin with 1% DNCB in the first phase and repeated application of 0.3% or 0.5% DNCB in the second phase. Animals of all groups increased their weight with no significant differences between the groups at the final day 29 (Figure 1a). Different degrees of inflammation and increased hair growth of the DNCB-treated skin were observed during the experiment (Figure S1). At day 15, the skin of the DNCB-treated mice showed the highest hlAD eczema severity which decreased in the further course of the experiment (Figure 1b). The final dermatitis score (DS) of each group was evaluated at the end of the experiment (Figure 1c). Both DNCB groups showed a mild DS of 1.6 ± 0.2 (0.3% DNCB) and 1.8 ± 0.4 (0.5% DNCB) at day 29, respectively.

### 2.2. Effects of DNCB on Secondary Lymphoid Organs

**Figure 2:**
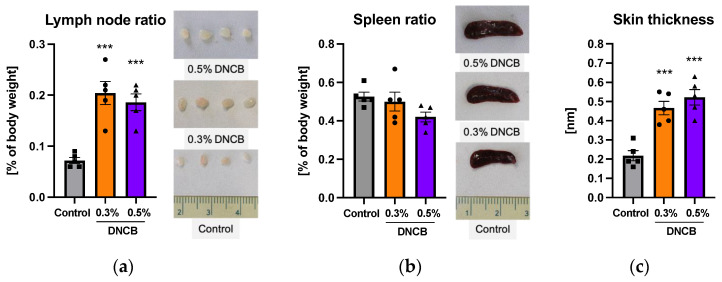
(**a**) Lymph node ratio and respective images; (**b**) Spleen ratio and respective images; (**c**) Skin thickness. Data are presented as the mean ± SEM (*n* = 5/group). Squares, dots and triangles represent one individual. *** *p* < 0.001 versus control.

Secondary lymphoid organs, such are lymph nodes or the spleen, are known to regulate antigen-driven lymphocyte maturation [25,26]. Significant increases in the weight and size of the lymph nodes compared to the control were detected (Figure 2a; *** *p* < 0.001). In contrast, these effects were not observed for the spleen (Figure 2b). Morphological changes in the DNCB-treated skin area were accompanied by a significantly increased skin thickness (Figure 2c; *** *p* < 0.001). In general, no differences in organ ratio or skin thickness were detected between the DNCB groups.

AD is known as a T-helper (TH) cell-mediated disease. Therefore, the TH cell population in splenocytes and lymphocytes was examined. We found no significant differences for the CD4^+^ and CD8^+^-cell populations between the groups (Figure S2).

### 2.3. Histological and Immunohistological Changes of DNCB-Treated Skin

**Figure 3:**
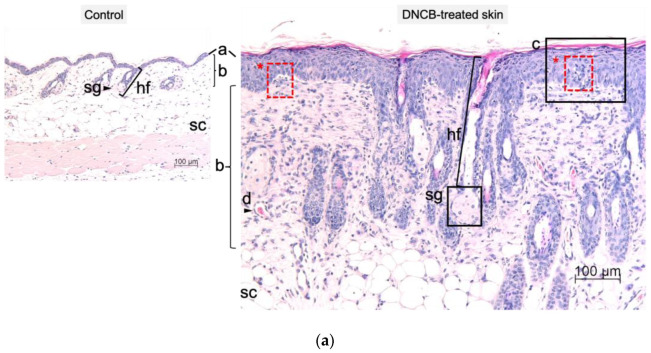

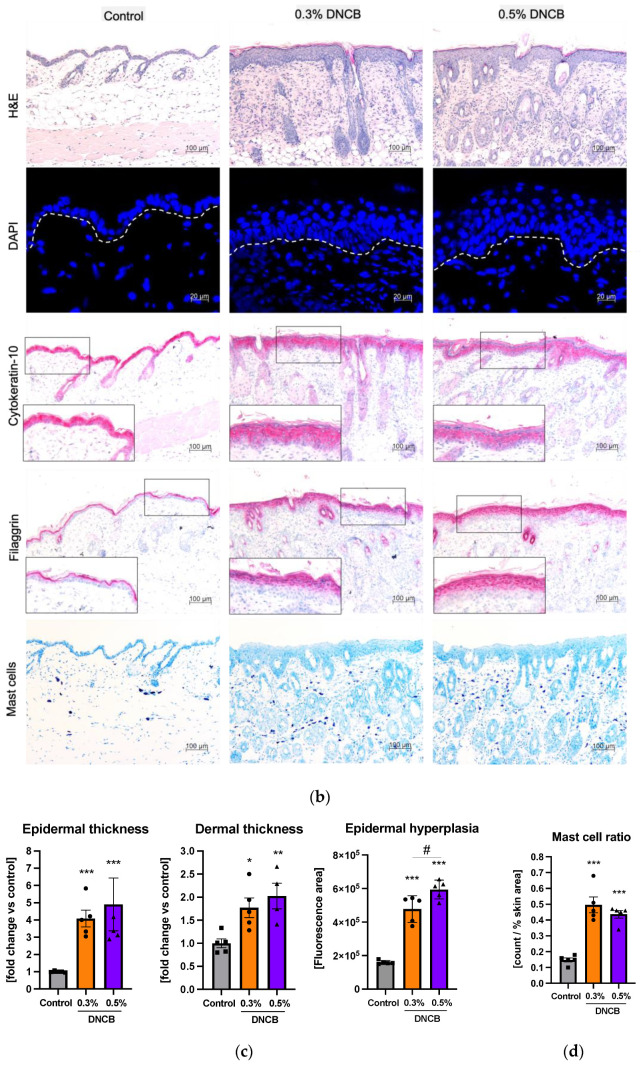
(**a**) Representative hematoxylin and eosin (H&E) staining of dorsal skin sections showing epidermis (a), dermis (b), subcutis (sc), hair follicle (hf), and sebaceous glands (sg). The DNCB-treated skin displayed thickening of the epidermis (a), altered dermal skin structure (b), acanthosis and hyperkeratosis (c), and blood vessels (d) as well as spongiosis (*). (**b**) Representative H&E-stained sections, fluorescence staining of cell nuclei, cytokeratin-10, and filaggrin and toluidine staining of the treated dorsal skin. Scale bar: 100 µm. (**c**) Evaluation of the epidermal and dermal thickness on H&E-stained sections and epidermal hyperplasia using stained cell nuclei. (**d**) Evaluation of mast cell infiltration into the skin. Data are presented as the mean ± SEM (*n* = 4–5/group). Squares, dots and triangles represent one individual. * *p* < 0.05, ** *p* < 0.01 and *** *p* < 0.001 versus control. # *p* < 0.05 between DNCB groups.

Significant morphological changes in skin thickness were confirmed by histological analysis (Figure 3a,b). Hematoxylin and eosin (H and E) staining of dorsal sections (Figure 3a,b, H&E) and the right ear (Figure S3) showed hyperplasia, hyperkeratosis, and spongiosis in both DNCB-treated groups. Epidermal and dermal skin thickness of the back as well as epidermal hyperplasia increased significantly in both DNCB-treated groups compared to the control group (Figure 3c; * *p* < 0.05, ** *p* < 0.01, *** *p* < 0.001). Epidermal hyperplasia was more pronounced in the 0.5% DNCB-treated group compared to the 0.3% DNCB-treated group (Figure 3c; # *p* < 0.05).

Immunohistochemical staining of the skin samples showed increased skin barrier proteins such as filaggrin and cytokeratin-10 in the DNCB-treated groups (Figure 3b, cytokeratin-10, filaggrin) while no differences were detected between the DNCB concentrations. An evaluation of immune cell infiltration into the skin showed a significantly increased number of mast cells in both DNCB groups compared to the control, with no difference between the DNCB groups (Figure 3d; *** *p* < 0.001).

### 2.4. Effects of DNCB on IgE and Histamine Concentrations

**Figure 4:**
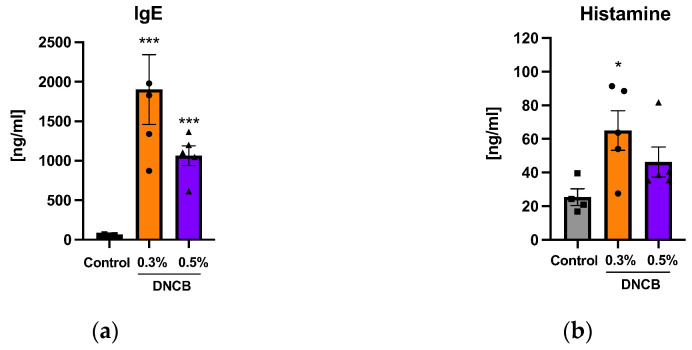
Measurement of (**a**) IgE and (**b**) histamine concentrations in plasma of DNCB-treated skin. Data are presented as the mean ± SEM (*n* = 4–5/group). Squares, dots and triangles represent one individual. * *p* < 0.05 and *** *p* < 0.001 versus control.

Significantly elevated IgE concentrations in the plasma were found in both DNCB-treated groups compared to the control on day 29, independent from the DNCB concentration (Figure 4a; *** *p* < 0.001). The histamine concentration in the plasma was significantly increased in the 0.3% DNCB-treated skin (Figure 4b; b; * *p* < 0.05). IL-5, IL-6, IL-4, IL-10, IL-13, and TNF-*α* in plasma samples were not detectable.

### 2.5. Effects of DNCB on Gene and Cytokine Expression and Lipid Mediator Formation

**Figure 5:**
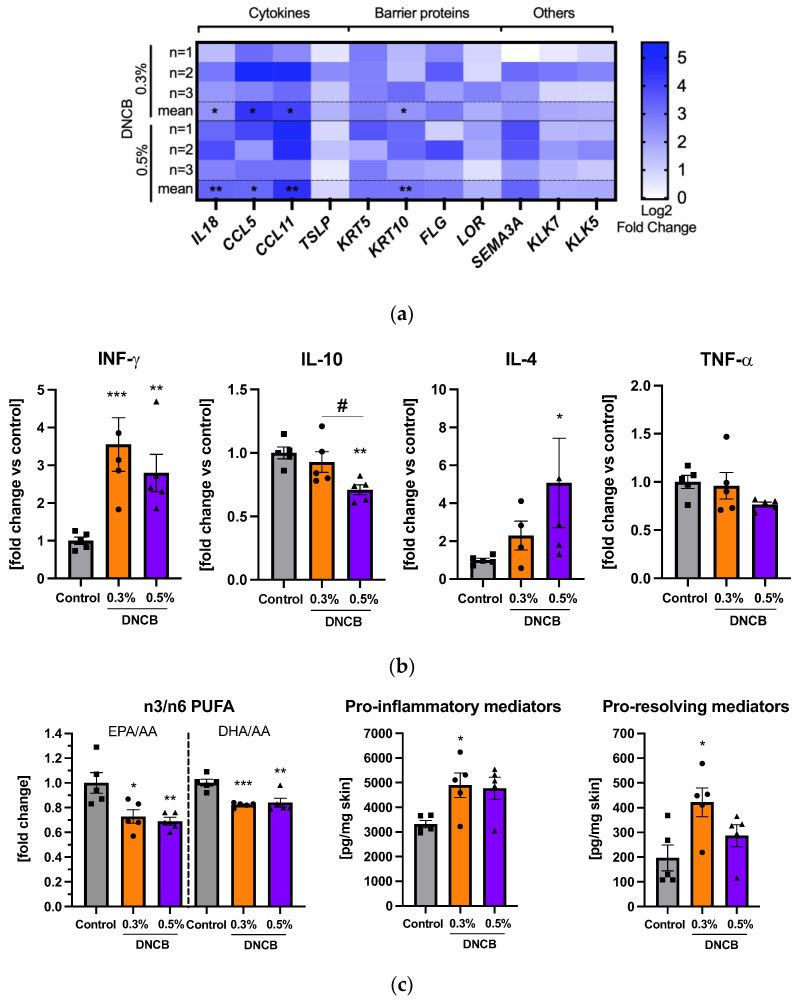
(**a**) Effects of DNCB on mRNA expression in skin. Heat maps present regulated genes compared to the control. Data are presented from individuals and as means (*n* = 3/group). (**b**) Measurement of cytokine expression in the skin. (**c**) Measurement of lipid mediator formation in the skin. Data present the ratio of omega-3 (n3) long-chain polyunsaturated fatty acids (PUFA) eicosapentaenoic (EPA) and docosahexaenoic (DHA) to the n6 PUFA arachidonic acid (AA), the sum of pro-inflammatory lipid mediators (PGD_2_, PGE_2_, PGF_2α_, TXB_2_, and LTB_4_), and the sum of pro-resolving lipid mediators (RvD5, PD1, MaR2, PDX, 17-HDHA, and 15-HEPE). Data are presented as the mean ± SEM (*n* = 4–5/group). Squares, dots and triangles represent one individual. * *p* < 0.05, ** *p* < 0.01 and *** *p* < 0.001 versus control. # *p* < 0.05 between DNCB groups.

Upregulation of mRNA expression of the pro-inflammatory cytokines *IL18*, *CCL5*, *CCL11,* thymic stromal lymphopoietin (*TSLP*), and cytokeratin-10 (*KRT10*) were evident in the DNCB-treated skin (Figure 5a). mRNA expression of skin barrier proteins, such as *KRT5*, filaggrin (*FLG*), loricrin (*LOR*), or other AD associated genes such as semaphorin 3A (*SEMA3A*) and kallikrein 5 and 7 (*KLK5*, *KLK7*), showed a trend to be upregulated. The pro-inflammatory cytokine interferon-γ (IFN-γ) was significantly increased in the DNCB-treated skin compared to the control (Figure 5b; ** *p* < 0.01, *** *p* < 0.001) while the anti-inflammatory cytokine IL-10 was significantly decreased in the 0.5% DNCB group compared to both the control group and the 0.3% DNCB- treated skin (Figure 5b; ** *p* < 0.01, # *p* < 0.05). The TH2 cell-related cytokine IL-4 was significantly elevated only in the 0.5% DNCB group (Figure 5b; * *p* < 0.05) whereas expression of TNF-α remained unchanged (Figure 5b). Other cytokines, such as IL-5, IL-17A, and IL-13 were below the detection limit.

Moreover, the formation of a broad spectrum of lipid mediators (LM) was measured in the treated skin (Figure S4). The ratio of omega-3 to -6 (n3/n6)-polyunsaturated fatty acids (PUFA), such as eicosapentaenoic (EPA) or docosahexaenoic (DHA) to arachidonic acid (AA), were significantly decreased in the DNCB groups compared to the control (Figure 5c; * *p* < 0.05, ** *p* < 0.01; and *** *p* < 0.001). The pro-inflammatory LM increased significantly in the 0.3% DNCB group (Figure 5c; * *p* < 0.05) with a similar effect in the 0.5% DNCB group compared to the control (Figure 5c; *p* = 0.054). Simultaneously, significantly elevated levels of pro-resolving LM were also detected in the 0.3% DNCB-treated group (Figure 5c; * *p* < 0.05).

### 2.6. Effects of DNCB on Cytokine Expression in Splenocytes

**Figure 6:**
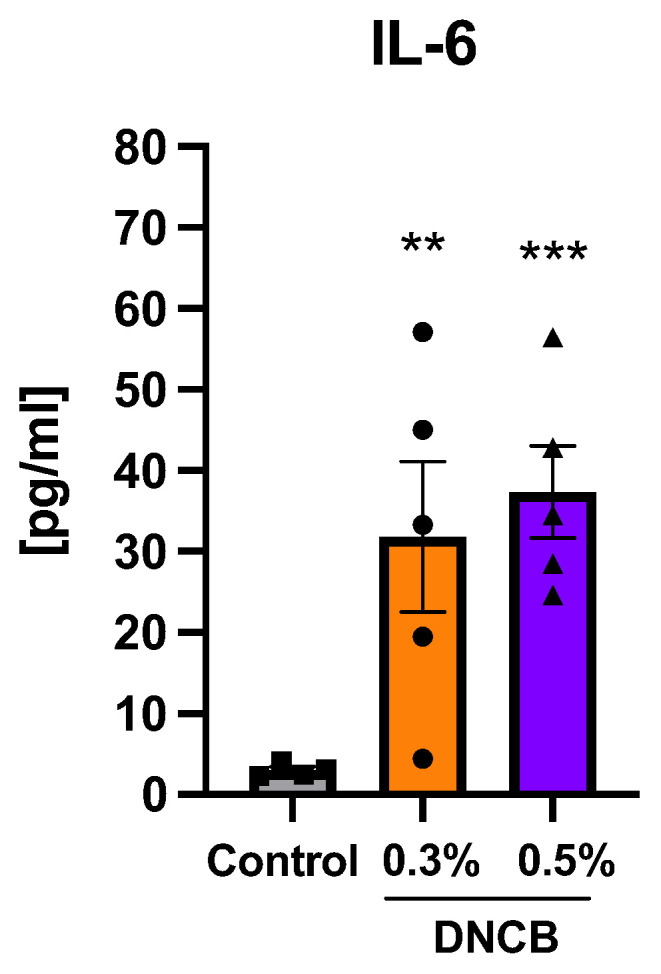
IL-6 expression from anti-CD3 and anti-CD28-activated splenocytes after 48 h of incubation. Data are presented as the mean ± SEM (*n* = 5/group). Squares, dots and triangles represent one individual. ** *p* < 0.01 and *** *p* < 0.001 versus control.

The pro-inflammatory cytokine IL-6 from isolated and activated splenocytes was significantly increased in both DNCB-treated groups compared to the control (Figure 6; ** *p* < 0.01 and *** *p* < 0.001).

## 3. Discussion

DNCB acts as an allergen-associated hapten that is picked up by local skin dendritic cells leading to an inflammatory response in the skin [27,28]. In this study, DNCB was applied to murine skin in the first phase (1% DNCB) and in the challenging phase (0.3% or 0.5% DNCB) to induce hlAD. During the study, different severity degrees of the eczema were acquired independently from the applied DNCB concentration which peaked on day 15 and declined by the end of the experiment. So far, this effect was only reported by a few studies [16,29,30] while other studies showed increased or equal severities [22,31,32,33,34] over the course of the experiment or endpoint scoring only [23,35,36,37]. In this study, the DNCB-treated skin showed markedly increased hair growth. A hair growth-inducing effect of the DNCB therapy was described in alopecia areata patients [38] but not for the hlAD model.

There are no official AD severity scores for murine skin in contrast to humans. Additionally, scarce information hampered the comparability of AD scoring between different studies. However, our result of the DS assigned the DNCB-treated lesions in this study to a human-like mild expression of AD severity.

A key characteristic of AD is the imbalance in immune reactions related to the TH cell response [3,4,39]. Naïve TH cells are present in the regional lymph nodes and polarized by migrated hapten-loaded skin resident antigen-presenting cells [40,41]. Only a few studies of DNCB models show data on morphological changes in lymphoid organs [17,21,23,29,42]. Although lymph node hypertrophy of the DNCB-treated mice was measured in this study, CD4^+^ or CD8^+^ cells were not altered in isolated lymphocytes. The spleen neither showed an increased weight nor an imbalance in the TH cell population but activated splenocytes presented a significant increase in IL-6, indicating an inflammatory response [43]. However, systemic inflammation with a focus on pro-inflammatory cytokines was not detectable in blood plasma. A previous study describes that a systemic reaction is lacking in mild expression of AD in contrast to moderate or severe degrees of severity [39]. Systemic inflammation markers in the serum were described in moderate to severe DNCB-induced hlAD skin lesions [18,29,44,45] which leads to the assumption that pro-inflammatory cytokine expression in plasma might have been elevated at day 15 of this experiment when the murine skin lesions revealed moderate hlAD. Likely, the DNCB treatment in our study induced a local effect in secondary lymphoid organs but did not lead to systemic inflammation at the end of the experiment.

The local inflammatory response in the treated skin area was proven by altered cytokine and mRNA expression of pro-inflammatory markers as well as LM formation. An upregulation of *CCL5* and *CCL11* mRNA expression, shown in this study, was also found in human mild AD with *CCL11* progressively increasing with the disease severity [39]. *KLK5* and *KLK7*, which were found to be elevated in the skin of AD patients [46,47,48], also showed a trend for upregulation in the DNCB-treated murine skin.

Furthermore, the DNCB treatment influenced the ratio of n3/n6 PUFAs in the skin while showing increased AA levels, similar to the skin from AD patients [49]. An inflammatory skin milieu was measured by summarizing pro-inflammatory LM such as leukotriene B isomers or prostaglandins (PGs). PGE_2_, a potent pro-inflammatory LM derived from AA by cyclooxygenase [50], is the main generated PG in the skin of DNCB-treated mice. Also, increased PGE_2_ levels were measured in lesional and non-lesional skin from AD patients, suggesting that PGE_2_ may be involved in AD pathogenesis [51]. Hong et al. [52] showed elevated levels of another important pro-inflammatory LM of the AA metabolism in the murine skin (12-hydroxyeicosatetraenoic acids) after seven weeks of DNCB treatment which were not found to be significantly increased in our study. Interestingly, the pro-resolving LM were also increased in the hlAD skin, indicating a tendency of counteracting the DNCB-induced inflammation by skin cells.

TH cells play a key role in the pathogenesis of AD while the pro-inflammatory cytokines IFN-γ and IL-4 are associated with specific functions [3,53]. A significant increase in IL-4 was only detected in the 0.5% DNCB group. IFN-γ was shown in both DNCB-treated groups and is known to have thickening effects in murine skin as it was observed in this study [54]. Epidermal thickening is seen in acute as well as in chronic lesions of human AD [3] and at all degrees of severity [39]. The dominance of the TH1-related cytokine IFN-γ is mainly associated with chronic lesions in human AD, while IL-4, a TH2 cytokine, is connected to acute lesions [3]. Our literature search shows that this classification is difficult for hlAD murine skin. Chen et al. [55] reports that TH2 cytokines are more prominent in early disease stages while Kitagaki et al. [56] shows that a prolonged antigen treatment leads to a shift from a TH1 to a TH2 cytokine response and a study on Stat6VT mice reveals a simultaneous increase in IL-4 and IFN-γ mRNA expression from the acute to chronic phase [57]. Since data are limited, an explicit assignment to an acute or chronic stage of the DNCB-treated skin lesions, as it is described for human AD, is not yet possible in this model. However, data suggest that the DNCB-induced hlAD model pictures a mixture of histologically acute (spongiosis [4]) and chronic lesions (marked epidermal thickening [3]) while IL-4 and IFN-γ seem to be both involved in the inflammatory response and skin abnormalities.

Skin barrier proteins play a crucial role in protecting skin from epidermal water loss and microbial, allergen, or chemical penetration [53]. We found increased mRNA levels of skin barrier proteins in the DNCB-treated skin. However, this effect cannot be clearly supported in an increased staining intensity per cell in the immunohistological slides. Nevertheless, an upregulation of filaggrin is described on an mRNA and protein level by Kim et al. [58]. Conversely, there are also studies that show a downregulation of filaggrin in moderate to severe lesions in the DNCB-induced hlAD models [23,59]. In human AD skin, the downregulation of skin barrier-associated genes is more characteristic for moderate to severe AD skin and they are less affected in mild expressions [7,60].

Elevated IgE levels are often measured in the hlAD model as a link to type I allergies [16,19,22,23,32,35,59]. In human AD, the presence of IgE distinguishes the extrinsic from the intrinsic type while extrinsic AD is triggered by allergens and is associated with a high serum IgE level [7,9]. The activation of mast cells is mediated by IgE antibodies followed by histamine release. The increased infiltration of mast cells into skin as well as elevated IgE and histamine concentrations in plasma reflect this allergic reaction cascade in our DNCB-induced hlAD model.

Taken together, our DNCB-induced hlAD model mainly depicts an extrinsic form with a mild disease severity. We identified basic criteria which are associated with mild eczema presented by local skin inflammation and abnormalities. To our knowledge, we are the first to measure a broad spectrum of pro-inflammatory LM and SPM formation, indicating an inflammatory response and, simultaneously, a regression in inflammation in the DNCB-treated skin of BALB/c mice. These findings are supported by the investigation of gene and cytokine expression to characterize the DNCB-treated skin of BALB/c mice. In summary, this study presents a comprehensive spectrum of parameters which shows a local inflammatory skin milieu. However, more data are needed to refer the disease pattern to an acute or chronic stage of human AD. In general, AD is a highly complex disease with several subtypes. To date, there is no murine AD model available that satisfactorily reflects the whole complex pathomechanism of human AD fully [14].

In conclusion, our study contributes to a better understanding of the DNCB-induced hlAD model itself as well as the mechanisms of DNCB-treatment in murine skin and thus to the evaluation of new treatment strategies and medications in the therapy of AD.

## 4. Materials and Methods

### 4.1. Chemicals

DNCB (≥99%; Cat. No. 237329), olive oil (Cat. No. 75343), and PBS (Cat. No. D8537) were purchased from Sigma-Aldrich (Darmstadt, Germany) and acetone from VWR international (Darmstadt, Germany; Cat. No. 270725). DNCB was dissolved in acetone and olive oil (3:1, *v*/*v*). DMSO was purchased from Carl Roth (Cat. No. A994.2; Karlsruhe, Germany).

### 4.2. Animals and Housing Conditions

Specific-opportunistic-pathogen-free (SOPF) BALB/cJRj (8-week-old, female; JanvierLabs, Saint Berthevin Cedex, France) were acclimatized for one week. All animals were housed under SOPF conditions according to the FELASA recommendations [61]. Pathogens were tested in a three-month frequency. All animals were housed in individually ventilated cages (Green Line 500, TecniPlast, Germany) and kept in groups of two or three per cage (temperature: 22 ± 2 °C; humidity: 55 ± 10%; 12:12 h light–dark cycle). The animals were fed with a standard rodent diet (Ssniff Spezialdiäten, Soest, Germany) while having access to food and autoclaved water *ad libitum.* Cages were enriched with poplar wood pellets, a cardboard tunnel, a wooden stick, and cotton rolls as the nesting material. Cages were changed once a week. None of the mice died during the experiment.

### 4.3. Induction of Atopic Dermatitis

**Figure 7:**
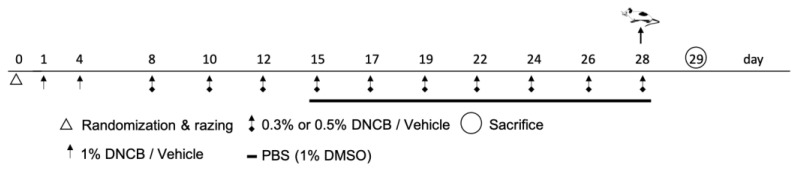
Time schedule of the DNCB-induced hlAD model on BALB/c mice by topical application.

After acclimatization, the animals were anesthetized with isoflurane, while their claws were shortened and a 5 cm^2^ area of the back of the mice was shaved (Aesculap Exacta GT416, B. Braun, Germany). The animals were randomized and divided in three groups with five mice each: the control group, 0.3% DNCB group, and 0.5% DNCB group. After one day of recovery, the animals from the DNCB groups were sensitized by applying 200 µL 1% DNCB on the back and 20 µL 1% DNCB behind the right ear by pipetting (Figure 7). The DNCB treatment was repeated at day 4. From day 8 onward, the animals were treated with 0.3% and 0.5% DNCB for three weeks (3x/week), respectively. The control group was treated with the vehicle (acetone:olive oil, 3:1, *v*/*v*). For application, the animals were fixed on the root of their tail. From day 15, all groups were treated with PBS (1% DMSO) approximately 1 h after DNCB treatment (200 µL) by slowly applying the solution to the inflamed skin and gently spreading it with a soft brush to simulate application of a vehicle solution for subsequent therapeutical target testing. One day after the last application (day 29) the animals were anesthetized with ketamine (100 mg/kg/body weight) and xylazine (15 mg/kg/body weight) and blood was taken with syringes containing 0.5 M EDTA.

The spleens and lymph nodes were weighted and photographed (Canon EOS 600D; Canon, Tokyo, Japan). The brightness of the organ images was equally adjusted using PowerPoint™ Microsoft 365^®^ (Redmond, WA, USA). Organs were stored overnight at 4 °C in PBS. Skin thickness of the DNCB treated area was recorded with a digital measuring stick (IP67, 150 mm, Mitutoyo, Japan). Skin samples for gene expression analysis were stored in RNA tissue protect reagent (Qiagen, Hilden, Germany) at −80 °C. Other skin samples were stored at −80 °C.

### 4.4. Scoring

Body weight was measured every week. The dermatitis score (DS) was calculated with regard to four disease specific symptoms based on the ‘severity scoring of AD on human skin‘ (SCORAD) [62]: dryness, erythema and redness, and edema and excoriation/crust formation. There is no official protocol available for a standard scoring of the eczema severity of murine skin. Therefore, estimation of DS from the literature was evaluated and used as a reference for scoring in this study. Each symptom was scored with 0 (none), 1 (mild < 20%), 2 (moderate 20–60%), and 3 (severe > 60%) during the experiment and on day 29 after shaving. The DS was defined as the sum of each individual symptom. The treated areas were photographed (Canon EOS 600D, Canon, Japan).

### 4.5. Organ Preparation and FACS Measurement

The spleens were crushed with the frosted sides of microscope slides and lysed with 2 mL red blood cell lysis buffer (ThermoFisher, Waltman, MA, USA). Cells were passed through a 70 µm nylon mesh to a volume of 7 mL, lysed for 10 min, and neutralized with 4 mL fluorescence activated cell sorting (FACS) buffer. The FACS buffer was based on PBS including 5% fetal calf serum (FCS) (Sigma Aldrich). Lymph nodes were crushed with 1 mL FACS buffer. Cells were passed through a 70 µm nylon mesh up to a volume of 5 mL. Cell suspensions were centrifugated (300× *g*, 10 min, 4 °C). The cells were resuspended in FACS buffer and passed through a 40 µm nylon mesh. Approximately 2 × 10^6^ cells of each sample were added into a 96-well plate. Cells were centrifuged (300× *g*, 10 min, 4 °C), washed, and incubated with an anti-CD16/CD32 Fc block (ThermoFisher). After 25 min, cells were stained with anti-CD45-PE, anti-CD3-APC, anti-CD4-FITC, and anti-CD8-PE-Cyanine7 (ThermoFisher) for 30 min at 4 °C. Cells were washed, fixed with fluorofix (BioLegend, Koblenz, Germany), and stored overnight at 4 °C. The next day, the cells were washed, resuspended with FACS buffer, and measured using flow cytometry (AttuneNxT, ThermoFisher).

### 4.6. Measurement of IgE and Histamine Concentration in Plasma

EDTA-blood was stored for 30 min at room temperature, centrifuged (300× *g*, 10 min), and plasma was aliquoted and stored at −80 °C. IgE was measured using a fluorescence-encoded bead-based assay on flow cytometry according to the manufacturer’s protocol (BioLegend). Histamine concentration in plasma samples was measured according to the manufacturers’ protocol (Immusmol, Bordeaux, France).

### 4.7. Cytokine Measurement in the Skin

Approximately 100 mg of skin tissue was washed with cold PBS and cut into small pieces and transferred into a 2 mL tube. In total, 900 µL of radioimmunoprecipitation assay buffer (Sigma Aldrich) was mixed 1:10 with a protease inhibitor cocktail (Sigma Aldrich) and 1 mM phenylmethylsulfonyl fluoride (Sigma Aldrich) was added to the tube. The skin was homogenized for 40 s with a tissue ruptor (Qiagen) on ice and incubated for 30 min. The samples were centrifuged (13,000× *g*, 10 min, 4 °C) and the supernatant was stored at −80 °C. The cytokines in the supernatant were measured with a fluorescence-encoded bead-based assay on flow cytometry according to the manufacturer´s protocol (LegendPlex, BioLegend).

### 4.8. Histological and Immunohistological Staining

The right ear and 6 mm ∅ of the dorsal skin were fixed with 4% phosphate buffered formaldehyde at room temperature (Carl Roth). Further preparation procedures are described in Appendix A. Cell nuclei were stained with 4′,6-diamidino-2-phenylindole (Sigma Aldrich). Microscopic assessment was carried out on the Axio Scope A.1 microscope using the DAPI-filter set to a wavelength from λ_ex_ = 358 nm (Carl Zeiss, Jena, Germany). Photographs of histological slides were taken with the digital camera AxioCam MRc (Carl Zeiss). An evaluation of the histological slides was conducted with ImageJ version v1.53t [63]. Mast cell infiltration into the skin was counted and calculated as the ratio to the relative area of the photographed skin.

### 4.9. RNA Isolation, cDNA Synthesis, and Quantitative Real-Time PCR

The RNA of skin samples was isolated and purified with the RNeasy Plus Universal Mini Kit (Qiagen) according to the manufacturer’s protocol. cDNA synthesis was conducted with the RT2 First Strand Kit (Qiagen). cDNA was added together with RT2 SYBR Green Mastermix (Qiagen) into a customized 384-well RT2 Profiler PCR Array plate (Qiagen) with 48 genes (Table S1). Real-time PCR was conducted with a LightCycler 480^®^ (Roche, Basel, Switzerland). The cycling conditions were programmed as recommended in the manufacturer’s protocol. The relative fold mRNA expression was calculated by the 2-ddCt-method using peptidylprolyl isomerase H as a housekeeping gene.

### 4.10. Lipid Mediator Metabololipidomics by UPLC-MS-MS

For each animal, 20 mg of skin were extracted in 400 µL PBS using a FastPrep-24 5G Lysis System (MP Biomedicals, Eschwege, Germany). Further preparation and method parameters are described in Appendix B. The mediators were measured using UPLC-MS/MS as described by Zhang et al. [64].

### 4.11. Splenocyte Cell Culture

Wells of a 12-well plate were coated at 37 °C for 2 h in a 5% CO_2_ atmosphere with 1µg/mL anti-CD3 (ThermoFisher). The coating supernatant was carefully removed and the isolated splenocytes were seeded at 3 × 10^6^ cells/mL (1 mL/well) into the coated 12-well plates using RPMI medium (Sigma Aldrich) containing 10% FCS, 2% PSG (Merck, Darmstadt, Germany), 2 mM GlutaMax (ThermoFisher), and 3 µg/mL anti-CD28 (ThermoFisher). The cells were further incubated for 48 h and the supernatant was collected and stored at −80 °C.

### 4.12. Statistical Analysis

Data are presented as mean ± standard error of the mean (SEM) using GraphPad version 9 (GraphPrism Software, San Diego, CA, USA). The determination of statistical significance was conducted on log-transformed data using one-way ANOVA followed by Tukey´s post hoc test. Statistical significance was tested with * *p* < 0.05, ** *p* < 0.01, and *** *p* < 0.001 versus the control or # *p* < 0.05 and ## *p* < 0.01 between the DNCB groups.

## Data Availability

All data underlying the results are available as part of the article and no additional source data are required. Further inquiries can be directed to the corresponding author.

## Supplementary Materials

The following supporting information can be downloaded at: https://www.mdpi.com/article/10.3390/ijms241512325/s1.

## Appendix A

Histological and immunohistological preparation was conducted with 4% phosphate buffered formaldehyde fixed skin samples. After 24 h, fixation samples were stored in PBS until embedding in paraffin blocks. Following this, samples were sliced with a thickness of 4 µm and dried at 60 °C. After dewaxing and hydration, the heat-induced epitope retrieval was conducted by incubation for 27 min in a steamer with TE-buffer (10 mM Tris Base, 1 mM EDTA, 0.05% Tween20; pH 9.0). Afterwards, the slides were washed two times for 5 min in 1xTBST buffer (50 mM M Tris Base, 150 mM NaCl, 0.025 M Triton X-100; pH 7.4). For a further 30 min, the slides were incubated in TBS with 1% Triton X-100. Nonspecific binding sites were blocked with UltraCruz^®^ Blocking Reagent (Santa Cruz Biotechnology, Dallas, TX, USA) for 30 min. Skin and ear samples were then blocked with the Avidin/Biotin Blocking System (BioLegend) accordingly. Immunohistochemical staining was conducted with one of the rabbit-anti-mouse primary antibodies filaggrin (BioLegend) or cytokeratin-10 (Sigma Aldrich) with a dilution of 1:2000 overnight at 4 °C. After washing with TBST two times for 5 min, the slides were incubated with the secondary biotin-labeled antibody (Jackson ImmunoResearch, Ely, UK) for 1 h. The slides were washed again and incubated with VECTASTAIN^®^ ABC-AP Kit (Vector Laboratories, Newark, CA, USA) for 30 min. After washing, the samples were treated with an alkaline phosphatase substrate (Abcam, Cambridges, USA) for 10 min followed by one washing step. Furthermore, the samples were counterstained for 30 s with haematoxylin Gill I (Merck), washed with tap water for 5 min, and covered with a coverslip and aquatex (Merck, Germany). Haematoxylin and eosin staining (H&E) of hydrated slides was performed with Haematoxylin Gill II (Merck) for 5 min; bluing was performed with tap water for 5 min and a 0.5% aqueus solution of Eosin Y (Merck) for 38 sec. They were then washed in ddH_2_O and ethanol to xylene and covered with a coverslip and Histofluid (Paul Marienfeld, Lauda-Königshofen, Germany).

Toluidine staining was conducted with 0.1% toluidine blue O (Sigma Aldrich) in 0.9% sodium chloride (pH 2.3). After deparaffinization and hydration with distilled water, the slides were stained for 2 min and washed with distilled water trice. Afterward, the slides were quickly dehydrated with 96% ethanol and 100% ethanol by dipping the slides 10 times into the alcohol, followed by a repeated clearance with xylene for 3 min and covering with a coverslip and Histofluid (Paul Marienfeld).

## Appendix B

After the skin samples were extracted, 800 µL of ice-cold methanol containing deuterated LM standards (200 nM d8-5S-HETE, d4-LTB_4_, d5-LXA_4_, d5-RvD2, d4-PGE_2_ and 10 µM d8-AA; Cayman Chemical/Biomol GmbH, Hamburg, Germany) was added. Samples were then kept at −20 °C for at least 60 min to allow protein precipitation. The extraction of LM in the supernatant was performed as recently published [64]. In brief, after centrifugation, (1200× *g*; 4 °C; 10 min) acidified H_2_O (9 mL; final pH = 3.5) was added and samples were extracted on solid phase cartridges (Sep-Pak^®^ Vac 6cc 500 mg/6 mL C18; Waters, Milford, MA, USA). Samples were loaded on the cartridges after equilibration with methanol followed by water. After washing with water and n-hexane, samples were eluted with methyl formate (6 mL). The solvent was fully evaporated using an evaporation system (TurboVap LV, Biotage, Uppsala, Sweden) and the residue was resuspended in 150 µL methanol/water (1:1, *v*/*v*) for UPLC-MS-MS analysis. LM were analyzed with an Acquity™ UPLC system (Waters, Milford, MA, USA) and a QTRAP 5500 Mass Spectrometer (ABSciex, Darmstadt, Germany) equipped with a Turbo V™ Source and electrospray ionization. LM were eluted using an ACQUITY UPLC^®^ BEH C18 column (1.7 µm, 2.1 mm × 100 mm; Waters, Eschborn, Germany) heated at 50 °C with a flow rate of 0.3 mL/min and a mobile phase consisting of methanol–water–acetic acid at a ratio of 42:58:0.01 (*v*/*v*/*v*) that was ramped to 86:14:0.01 (*v*/*v*/*v*) over 12.5 min and then to 98:2:0.01 (*v*/*v*/*v*) for 3 min [65]. The QTRAP 5500 was run in a negative ionization mode using scheduled multiple reaction monitoring (MRM) coupled with information-dependent acquisition. The scheduled MRM window was 60 s and optimized LM parameters were adopted [65] with a curtain gas pressure of 35 psi. The retention time and at least six diagnostic ions for each LM were confirmed by means of an external standard for each and every LM (Cayman Chemical/Biomol GmbH, Hamburg, Germany). Quantification was achieved by calibration curves for each LM. Linear calibration curves were obtained for each LM and gave r2 values of 0.998 or higher. The limit of detection for each targeted LM was determined as described [65].

## References

[1] Asher M.I., Montefort S., Björkstén B., Lai C.K., Strachan D.P., Weiland S.K., Williams H. (2006). Worldwide Time Trends in the Prevalence of Symptoms of Asthma, Allergic Rhinoconjunctivitis, and Eczema in Childhood: ISAAC Phases One and Three Repeat Multicountry Cross-Sectional Surveys. Lancet.

[2] Nutten S. (2015). Atopic Dermatitis: Global Epidemiology and Risk Factors. Ann. Nutr. Metab..

[3] Gittler J.K., Shemer A., Suárez-Fariñas M., Fuentes-Duculan J., Gulewicz K.J., Wang C.Q.F., Mitsui H., Cardinale I., de Guzman Strong C., Krueger J.G. (2012). Progressive Activation of TH2/TH22 Cytokines and Selective Epidermal Proteins Characterizes Acute and Chronic Atopic Dermatitis. J. Allergy Clin. Immunol..

[4] Guttman-Yassky E., Nograles K.E., Krueger J.G. (2011). Contrasting Pathogenesis of Atopic Dermatitis and Psoriasis—Part I: Clinical and Pathologic Concepts. J. Allergy Clin. Immunol..

[5] Burgess J.A., Dharmage S.C., Byrnes G.B., Matheson M.C., Gurrin L.C., Wharton C.L., Johns D.P., Abramson M.J., Hopper J.L., Walters E.H. (2008). Childhood Eczema and Asthma Incidence and Persistence: A Cohort Study from Childhood to Middle Age. J. Allergy Clin. Immunol..

[6] Dharmage S.C., Lowe A.J., Matheson M.C., Burgess J.A., Allen K.J., Abramson M.J. (2014). Atopic Dermatitis and the Atopic March Revisited. Allergy.

[7] Martel B.C., Litman T., Hald A., Norsgaard H., Lovato P., Dyring-Andersen B., Skov L., Thestrup-Pedersen K., Skov S., Skak K. (2016). Distinct Molecular Signatures of Mild Extrinsic and Intrinsic Atopic Dermatitis. Exp. Dermatol..

[8] Tokura Y. (2010). Extrinsic and Intrinsic Types of Atopic Dermatitis. J. Dermatol. Sci..

[9] Novak N., Bieber T. (2003). Allergic and Nonallergic Forms of Atopic Diseases. J. Allergy Clin. Immunol..

[10] Bawany F., Northcott C.A., Beck L.A., Pigeon W.R. (2021). Sleep Disturbances and Atopic Dermatitis: Relationships, Methods for Assessment, and Therapies. J. Allergy Clin. Immunol. Pract..

[11] Schonmann Y., Mansfield K.E., Hayes J.F., Abuabara K., Roberts A., Smeeth L., Langan S.M. (2020). Atopic Eczema in Adulthood and Risk of Depression and Anxiety: A Population-Based Cohort Study. J. Allergy Clin. Immunol. Pract..

[12] Thyssen J.P., Hamann C.R., Linneberg A., Dantoft T.M., Skov L., Gislason G.H., Wu J.J., Egeberg A. (2018). Atopic Dermatitis Is Associated with Anxiety, Depression, and Suicidal Ideation, but not with Psychiatric Hospitalization or Suicide. Allergy.

[13] Jin H., He R., Oyoshi M., Geha R.S. (2009). Animal Models of Atopic Dermatitis. J. Investig. Dermatol..

[14] Gilhar A., Reich K., Keren A., Kabashima K., Steinhoff M., Paus R. (2021). Mouse Models of Atopic Dermatitis: A Critical Reappraisal. Exp. Dermatol..

[15] Pickard C., Smith A.M., Cooper H., Strickland I., Jackson J., Healy E., Friedmann P.S. (2007). Investigation of Mechanisms Underlying the T-Cell Response to the Hapten 2,4-Dinitrochlorobenzene. J. Investig. Dermatol..

[16] Jin W., Huang W., Chen L., Jin M., Wang Q., Gao Z., Jin Z. (2018). Topical Application of JAK1/JAK2 Inhibitor Momelotinib Exhibits Significant Anti-Inflammatory Responses in DNCB-Induced Atopic Dermatitis Model Mice. Int. J. Mol. Sci..

[17] Hou D.-D., Di Z.-H., Qi R.-Q., Wang H.-X., Zheng S., Hong Y.-X., Guo H., Chen H.-D., Gao X.-H. (2017). Sea Buckthorn (Hippophaë rhamnoides L.) Oil Improves Atopic Dermatitis-Like Skin Lesions via Inhibition of NF-κB and STAT1 Activation. Skin Pharmacol. Physiol..

[18] Kang B.-K., Kim M.-J., Kim K.-B.-W.-R., Ahn D.-H. (2016). In Vivo and In Vitro Inhibitory Activity of an Ethanolic Extract of *Sargassum fulvellum* and Its Component Grasshopper Ketone on Atopic Dermatitis. Int. Immunopharmacol..

[19] Kim S.R., Choi H.-S., Seo H.S., Ku J.M., Hong S.H., Yoo H.H., Shin Y.C., Ko S.-G. (2014). Oral Administration of Herbal Mixture Extract Inhibits 2,4-Dinitrochlorobenzene-Induced Atopic Dermatitis in BALB/c Mice. Mediat. Inflamm..

[20] Han S.-C., Kang G.-J., Ko Y.-J., Kang H.-K., Moon S.-W., Ann Y.-S., Yoo E.-S. (2012). Fermented Fish Oil Suppresses T helper 1/2 Cell Response in a Mouse Model of Atopic Dermatitis via Generation of CD4+CD25+Foxp3+ T Cells. BMC Immunol..

[21] Heo J.H., Heo Y., Lee H.J., Kim M., Shin H.Y. (2018). Topical Anti-Inflammatory and Anti-Oxidative Effects of porcine Placenta Extracts on 2,4-dinitrochlorobenzene-Induced Contact Dermatitis. BMC Complement Altern. Med..

[22] Wang Y., Zhang P., Zhang J., Hong T. (2022). Inhibitory Effect of Bisdemethoxycurcumin on DNCB-Induced Atopic Dermatitis in Mice. Molecules.

[23] Jang S., Ohn J., Kim J.W., Kang S.M., Jeon D., Heo C.Y., Lee Y.-S., Kwon O., Kim K.H. (2020). Caffeoyl–Pro–His Amide Relieve DNCB-Induced Atopic Dermatitis-Like Phenotypes in BALB/c mice. Sci. Rep..

[24] Council of Europe (2010). Directive 2010/63/EU of the European Parliament and of the Council of 22 September 2010 on the Protection of Animals Used for Scientific Purposes.

[25] Pabst R. (2007). Plasticity and Heterogeneity of Lymphoid Organs. Immunol. Lett..

[26] Lewis S.M., Williams A., Eisenbarth S.C. (2019). Structure and Function of the Immune System in the Spleen. Sci. Immunol..

[27] Carr M.M., Botham P.A., Gawkrodger D.J., Mcvittie E., Ross J.A., Stewart I.C., Hunter J.A.A. (1984). Early Cellular Reactions Induced by Dinitrochlorobenzene in Sensitized Human Skin. Br. J. Dermatol..

[28] Gawkrodger D.J., Haftek M., Botham P.A., Carr M.M., Spencer M.-J., Ross J.A., Hunter J.A.A., Thivolet J. (1989). The Hapten in Contact Hypersensitivity to Dinitrochlorobenzene: Immunoelectron Microscopic and Immunofluorescent Studies. Dermatology.

[29] Kang J.A., Song H.-Y., Byun E.-H., Ahn N.-G., Kim H.-M., Nam Y.R., Lee G.H., Jang B.-S., Choi D.S., Lee D.-E. (2018). Gamma-Irradiated Black Ginseng Extract Inhibits Mast Cell Degranulation and Suppresses Atopic Dermatitis-Like Skin Lesions in Mice. Food Chem. Toxicol..

[30] Kim H.W., Ju D.B., Kye Y.-C., Ju Y.-J., Kim C.G., Lee I.K., Park S.-M., Choi I.S., Cho K.K., Lee S.H. (2020). Galectin-9 Induced by Dietary Probiotic Mixture Regulates Immune Balance to Reduce Atopic Dermatitis Symptoms in Mice. Front. Immunol..

[31] Kim Y.-E., Cho N., Cheon S., Kim K.K. (2017). Bortezomib, A Proteasome Inhibitor, Alleviates Atopic Dermatitis by Increasing Claudin 1 Protein Expression. Biochem. Biophys. Res. Commun..

[32] Li Y., Chen L., Du Y., Huang D., Han H., Dong Z. (2016). Fluoxetine Ameliorates Atopic Dermatitis-Like Skin Lesions in BALB/c Mice through Reducing Psychological Stress and Inflammatory Response. Front. Pharmacol..

[33] Purushothaman B., Arumugam P., Kulsi G., Song J.M. (2018). Design, Synthesis, and Biological Evaluation of novel Catecholopyrimidine Based PDE4 Inhibitor for the Treatment of Atopic Dermatitis. Eur. J. Med. Chem..

[34] Peng G., Mu Z., Cui L., Liu P., Wang Y., Wu W., Han X. (2018). Anti-IL-33 Antibody Has a Therapeutic Effect in an Atopic Dermatitis Murine Model Induced by 2, 4-Dinitrochlorobenzene. Inflammation.

[35] Wu C.-S., Lin S.-C., Li S., Chiang Y.-C., Bracci N., Lehman C.W., Tang K.-T., Lin C.-C. (2020). Phloretin Alleviates Dinitrochlorobenzene-Induced Dermatitis in BALB/c Mice. Int. J. Immunopathol. Pharmacol..

[36] Chang Y.-S., Tsai C.-C., Yang P.-Y., Tang C.-Y., Chiang B.-L. (2022). Topical Melatonin Exerts Immunomodulatory Effect and Improves Dermatitis Severity in a Mouse Model of Atopic Dermatitis. Int. J. Mol. Sci..

[37] Bak D.-H., Lee E., Lee B.C., Choi M.J., Kwon T.-R., Hong J., Mun S., Lee K., Kim S., Na J. (2019). Therapeutic Potential of Topically Administered γ-AlOOH on 2,4-Dinitrochlorobenzene-Induced Atopic Dermatitis-Like Lesions in Balb/c Mice. Exp. Dermatol..

[38] Happle R., Echternacht K. (1977). Induction of Hair Growth in Alopecia Areata with D.N.C.B. Lancet.

[39] He H., Del Duca E., Diaz A., Kim H.J., Gay-Mimbrera J., Zhang N., Wu J., Beaziz J., Estrada Y., Krueger J.G. (2021). Mild Atopic Dermatitis Lacks Systemic Inflammation and Shows Reduced Nonlesional Skin Abnormalities. J. Allergy Clin. Immunol..

[40] Biedermann T., Skabytska Y., Kaesler S., Volz T. (2015). Regulation of T Cell Immunity in Atopic Dermatitis by Microbes: The Yin and Yang of Cutaneous Inflammation. Front. Immunol..

[41] Rafei-Shamsabadi D.A., Klose C.S.N., Halim T.Y.F., Tanriver Y., Jakob T. (2019). Context Dependent Role of Type 2 Innate Lymphoid Cells in Allergic Skin Inflammation. Front. Immunol..

[42] Bai X.-Y., Liu P., Chai Y.-W., Wang Y., Ren S.-H., Li Y.-Y., Zhou H. (2020). Artesunate Attenuates 2, 4-Dinitrochlorobenzene-Induced Atopic Dermatitis by Down-Regulating Th17 Cell Responses in BALB/c Mice. Eur. J. Pharmacol..

[43] Yang H.-L., Yang T.-Y., Gowrisankar Y.V., Liao C.-H., Liao J.-W., Huang P.-J., Hseu Y.-C. (2020). Suppression of LPS-Induced Inflammation by Chalcone Flavokawain A through Activation of Nrf2/ARE-Mediated Antioxidant Genes and Inhibition of ROS/NF *κ* B Signaling Pathways in Primary Splenocytes. Oxidative Med. Cell. Longev..

[44] Lin G., Gao S., Cheng J., Li Y., Shan L., Hu Z. (2016). 1 **β**-Hydroxyalantolactone, A Sesquiterpene Lactone from *Inula japonica*, Attenuates Atopic Dermatitis-Like Skin Lesions Induced by 2,4-Dinitrochlorobenzene in the Mouse. Pharm. Biol..

[45] Yu H., Li H., Li Y., Li M., Chen G. (2017). Effect of Isoliquiritigenin for the Treatment of Atopic Dermatitis-Like Skin Lesions in Mice. Arch. Dermatol. Res..

[46] Komatsu N., Saijoh K., Kuk C., Liu A.C., Khan S., Shirasaki F., Takehara K., Diamandis E.P. (2007). Human tissue Kallikrein Expression in the Stratum Corneum and Serum of Atopic Dermatitis Patients. Exp. Dermatol..

[47] Guo C.J., Mack M.R., Oetjen L.K., Trier A.M., Council M.L., Pavel A.B., Guttman-Yassky E., Kim B.S., Liu Q. (2020). Kallikrein 7 Promotes Atopic Dermatitis-Associated Itch Independently of Skin Inflammation. J. Investig. Dermatol..

[48] Zhu Y., Underwood J., Macmillan D., Shariff L., O’Shaughnessy R., Harper J.I., Pickard C., Friedmann P.S., Healy E., Di W.-L. (2017). Persistent Kallikrein 5 Activation Induces Atopic Dermatitis-Like Skin Architecture Independent of PAR2 Activity. J. Allergy Clin. Immunol..

[49] Töröcsik D., Weise C., Gericke J., Szegedi A., Lucas R., Mihaly J., Worm M., Rühl R. (2019). Transcriptomic and Lipidomic Profiling of Eicosanoid/Docosanoid Signalling in Affected and Non-Affected Skin of Human Atopic Dermatitis Patients. Exp. Dermatol..

[50] Funk C.D. (2001). Prostaglandins and Leukotrienes: Advances in Eicosanoid Biology. Science.

[51] Fogh K., Herlin T., Kragballe K. (1989). Eicosanoids in Skin of Patients with Atopic Dermatitis: Prostaglandin E and Leukotriene B are Present in Biologically Active Concentrations. J. Allergy Clin. Immunol..

[52] Hong S.-H., Han J.E., Ko J.-S., Do S.H., Lee E.H., Cho M.-H. (2015). Quantitative Determination of 12-Hydroxyeicosatetraenoic Acids by Chiral Liquid Chromatography Tandem Mass Spectrometry in a Murine Atopic Dermatitis Model. J. Vet. Sci..

[53] Bieber T. (2010). Atopic Dermatitis. Ann. Dermatol..

[54] Spergel J.M., Mizoguchi E., Oettgen H., Bhan A.K., Geha R.S. (1999). Roles of TH1 and TH2 Cytokines in a Murine Model of Allergic Dermatitis. J. Clin. Invest..

[55] Chen L., Martinez O., Overbergh L., Mathieu C., Prabhakar B.S., Chan L.S. (2004). Early Up-Regulation of Th2 Cytokines and Late Surge of Th1 Cytokines in an Atopic Dermatitis Model. Clin. Exp. Immunol..

[56] Kitagaki H., Ono N., Hayakawa K., Kitazawa T., Watanabe K., Shiohara T. (1997). Repeated Elicitation of Contact Hypersensitivity Induces A Shift in Cutaneous Cytokine Milieu from A T Helper Cell Type 1 to A T Helper Cell Type 2 Profile. J. Immunol..

[57] DaSilva-Arnold S.C., Thyagarajan A., Seymour L.J., Yi Q., Bradish J.R., Al-Hassani M., Zhou H., Perdue N.J., Nemeth V., Krbanjevic A. (2018). Phenotyping Acute and Chronic Atopic Dermatitis-Like Lesions in Stat6VT Mice Identifies a Role for IL-33 in Disease Pathogenesis. Arch. Dermatol. Res..

[58] Kim J., Lee J., Shin S., Cho A., Heo Y. (2018). Molecular Mechanism of Atopic Dermatitis Induction Following Sensitization and Challenge with 2,4-Dinitrochlorobenzene in Mouse Skin Tissue. Toxicol. Res..

[59] An H.-J., Kim J.-Y., Kim W.-H., Gwon M.-G., Gu H.M., Jeon M.J., Han S.-M., Pak S.C., Lee C.-K., Park I.S. (2018). Therapeutic Effects of Bee Venom and Its Major Component, Melittin, on Atopic Dermatitis In Vivo And In Vitro: Effects of Bee Venom and Melittin on Atopic Eczema. Br. J. Pharmacol..

[60] Mlitz V., Latreille J., Gardinier S., Jdid R., Drouault Y., Hufnagl P., Eckhart L., Guinot C., Tschachler E. (2012). Impact of Filaggrin Mutations on Raman Spectra and Biophysical Properties of the Stratum Corneum in Mild to Moderate Atopic Dermatitis: Raman Profiling of Mild to Moderate Atopic Dermatitis. J. Eur. Acad. Dermatol. Venereol..

[61] Mähler Convenor M., Berard M., Feinstein R., Gallagher A., Illgen-Wilcke B., Pritchett-Corning K., Raspa M., FELASA Working Group on Revision of Guidelines for Health Monitoring of Rodents and Rabbits (2014). FELASA Recommendations for the Health Monitoring of Mouse, Rat, Hamster, Guinea Pig and Rabbit Colonies in Breeding and Experimental Units. Lab. Anim..

[62] European Task Force on Atopic Dermatitis (1993). Severity Scoring of Atopic Dermatitis: The SCORAD Index. Dermatology.

[63] Schindelin J., Arganda-Carreras I., Frise E., Kaynig V., Longair M., Pietzsch T., Preibisch S., Rueden C., Saalfeld S., Schmid B. (2012). Fiji: An Open-Source Platform for Biological-Image Analysis. Nat. Methods.

[64] Zhang K., Pace S., Jordan P.M., Peltner L.K., Weber A., Fischer D., Hofstetter R.K., Chen X., Werz O. (2021). Beneficial Modulation of Lipid Mediator Biosynthesis in Innate Immune Cells by Antirheumatic Tripterygium wilfordii Glycosides. Biomolecules.

[65] Werner M., Jordan P.M., Romp E., Czapka A., Rao Z., Kretzer C., Koeberle A., Garscha U., Pace S., Claesson H. (2019). Targeting Biosynthetic Networks of the Proinflammatory and Proresolving Lipid Metabolome. FASEB J..

