# Supplementary material for: Establishment and Characterization of Mild Atopic Dermatitis in the DNCB-Induced Mouse Model
Source: Int J Mol Sci. 2023 Aug 1;24(15):12325. doi: 10.3390/ijms241512325 (PMC10418750; doi:10.3390/ijms241512325)
Supplement: Supplementary file 1 [file ijms-24-12325-s001.zip › ijms-2538650-supplementary.pdf]

Supplement to the manuscript:

Establishment and Characterization of Mild Atopic Dermatitis in the DNCB-Induced Mouse Model

Rebecca Riedl [1,2], Annika Kühn [2], Denise Rietz [1], Betty Hebecker [2,3], Karl-Gunther Glowalla [4], Lukas K. Peltner [5], Paul M. Jordan [5,6], Oliver Werz [5,6], Stefan Lorkowski [2,3], Cornelia Wiegand [1] and Maria Wallert [2,3,*]

1 University Hospital Department of Dermatology Jena, Dermatological Research Laboratory, 07747 Jena, Germany; rebecca.riedl@med.uni-jena.de (R.R.); denise.rietz@med.uni-jena.de (D.R.); c.wiegand@med.uni-jena.de (C.W.)

2 Department of Nutritional Biochemistry and Physiology, Institute of Nutritional Science, Friedrich Schiller University, 07743 Jena, Germany; annika.kuehn@uni-jena.de (A.K.); betty.hebecker@uni-jena.de (B.H.); stefan.lorkowski@uni-jena.de (S.L.)

3 Competence Cluster for Nutrition and Cardiovascular Health (nutriCARD) Halle-Jena-Leipzig, 07743 Jena, Germany

4 Service Unit Experimental Biomedicine, Friedrich Schiller University, 07745 Jena, Germany; karl-gunther.glowalla@uni-jena.de

5 Department of Pharmaceutical/Medicinal Chemistry, Institute of Pharmacy, Friedrich Schiller University, 07743 Jena, Germany; lukas.klaus.peltner@uni-jena.de (L.K.P.); paul.jordan@uni-jena.de (P.M.J.); oliver.werz@uni-jena.de (O.W.)

6 Jena Center for Soft Matter (JCSM), Friedrich Schiller University, 07743 Jena, Germany

* Correspondence: maria.wallert@uni-jena.de; Tel.: +49-3641-9-49726

Supplement

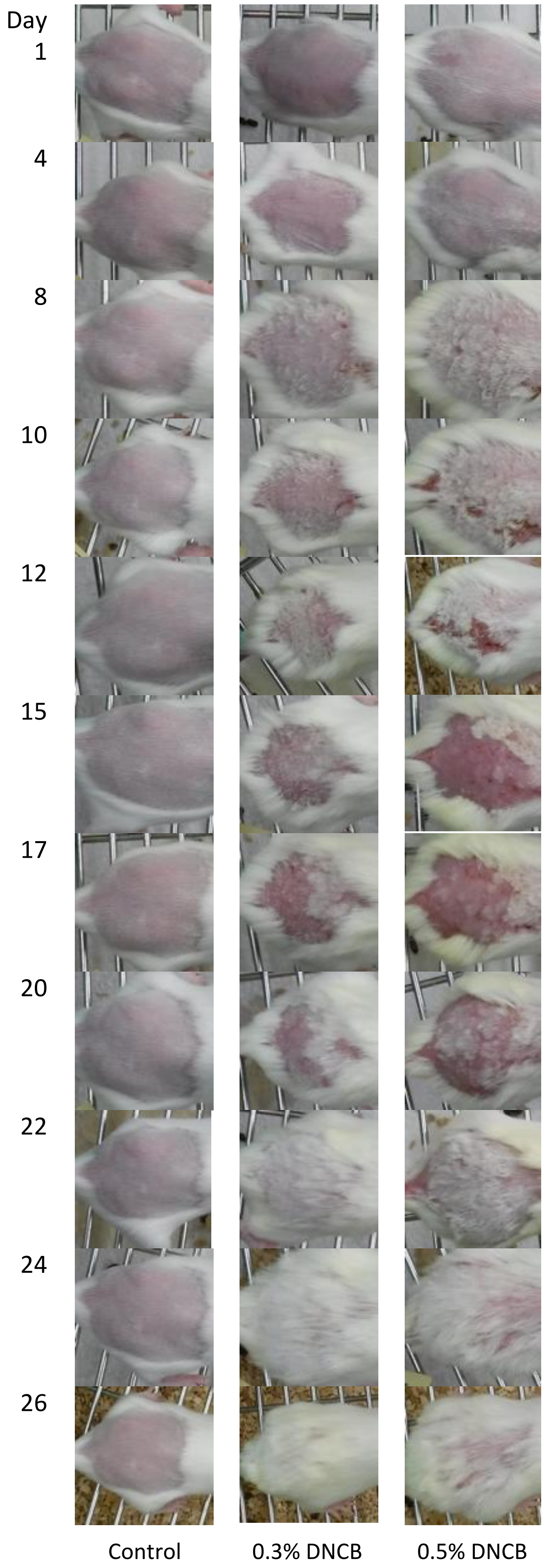

Figure S1. Representative images of the dorsal murine skin during the experiment. Images ware taken before DNCB or vehicle application.

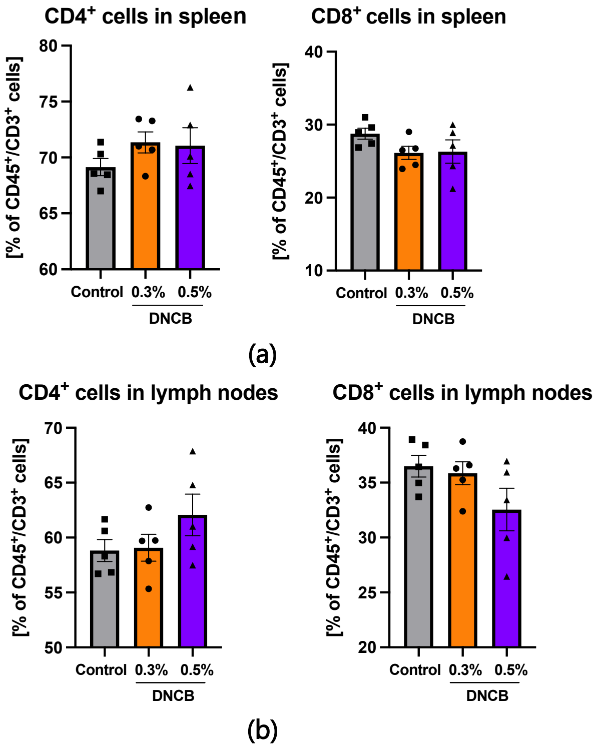

Figure S2. CD4+ and CD8+ cells in CD45+/CD3+ (**a**) isolated splenocytes and (**b**) lymphocytes at the end of the experiment. Data are presented as the mean ± SEM (n=5/group). Squares, dots and triangles represent one individual.

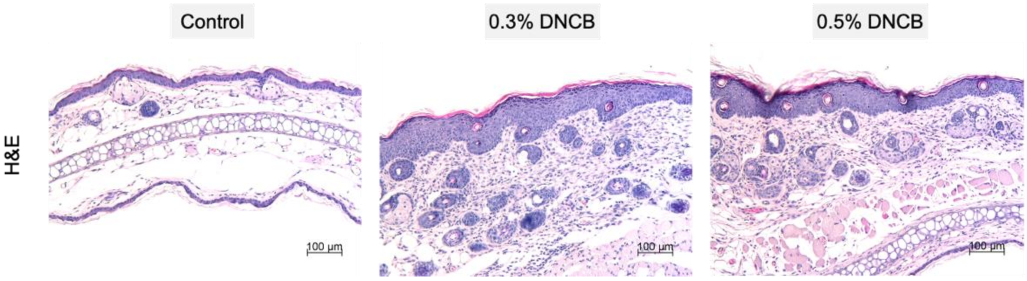

Figure S3. Representative hematoxylin and eosin (H&E) staining of the right ear. Scale bar: 100 µm.

0 — 1 — 2		control			0.3% DNCB				0.5% DNCB			
		mean		SEM	mean		SEM	f	mean		SEM	f
SPMs	RvD5	3.0	±	0.7	12	±	2.5	4.00	6.4	±	1.2	2.13
	PD1	0.7	±	0.3	3.7	±	0.7	5.29	2.3	±	0.4	3.29
	PDX	3.3	±	0.9	16	±	3.0	4.85	7.4	±	1.7	2.24
	MaR2	1.6	±	0.4	6.6	±	1.1	4.13	5.0	±	1.0	3.13
12-/15-LOX products	17-HDHA	136	±	30	331	±	44	2.43	222	±	34	1.63
	15-HEPE	20	±	5.4	53	±	9.6	2.65	44	±	7.8	2.20
	15-HETE	180	±	23	459	±	45	2.55	323	±	48	1.79
	14-HDHA	346	±	72	762	±	81	2.20	494	±	82	1.43
	12-HEPE	94	±	20	82	±	11	0.87	77	±	5.8	0.82
	12-HETE	1830	±	212	2441	±	295	1.33	2005	±	168	1.10
5-LOX products	7-HDHA	3.3	±	0.4	4.6	±	1.1	1.39	2.7	±	0.2	0.82
	5-HEPE	1.8	±	0.2	1.4	±	0.1	0.78	1.1	±	0.1	0.61
	5-HETE	46	±	5.7	56	±	7.6	1.22	38	±	2.7	0.83
	t-LTB$_4$	14	±	2.4	37	±	4.4	2.64	29	±	3.9	2.07
	et-LTB$_4$	18	±	3.3	42	±	5.8	2.33	35	±	4.6	1.94
	LTB$_4$	n.d.	±	-	n.d.	±	-	1.00	n.d.	±	-	1.00
COX products	PGD$_2$	1364	±	75	1165	±	114	0.85	1091	±	124	0.80
	PGE$_2$	1664	±	99	3265	±	394	1.96	3269	±	301	1.96
	PGF$_{2\alpha}$	157	±	6.4	230	±	30	1.46	236	±	40	1.50
	TXB$_2$	77	±	9.0	157	±	19	2.04	112	±	7.9	1.45
other	13-HDHA	41	±	3.7	40	±	5.3	0.98	36	±	7.4	0.88
	10-HDHA	16	±	3.9	31	±	5.3	1.94	18	±	3.6	1.13
	4-HDHA	6.0	±	0.8	6.7	±	1.1	1.12	4.7	±	0.4	0.78
	11-HEPE	3.1	±	0.3	4.3	±	0.4	1.39	4.2	±	0.8	1.35
	11-HETE	196	±	12	260	±	14	1.33	225	±	35	1.15
	8-HETE	91	±	23	225	±	41	2.47	123	±	22	1.35
	9-HODE	1252	±	68	1180	±	120	0.94	976	±	124	0.78
	13-HODE	1437	±	118	2049	±	271	1.43	1588	±	274	1.11
	5,15-diHETE	15	±	2.4	37	±	5.5	2.47	23	±	4.6	1.53
	5,12S-diHETE	19	±	3.3	47	±	7.0	2.47	33	±	7.4	1.74
PUFA	AA	34124	±	4420	56278	±	8018	1.65	37634	±	3056	1.10
	EPA	3437	±	597	4438	±	854	1.29	2745	±	308	0.80
	DHA	12377	±	1539	16961	±	2524	1.37	11693	±	1516	0.94

Figure S4. Effects of DNCB treatment on lipid mediator formation in murine dorsal skin. Data are presented in pg/mg as means ± SEM and fold change (f) to control group (n=5/ group).

Table S1. Commercially validated primers used with a customized 384-well RT2 PCR Array Plate (Qiagen)

Gene name	GeneGlobe ID (Qiagen)
PPIH	QT01165318
IL18	QT00171129
CCL5	QT01747165
CCL11	QT00114275
TSLP	QT00198261
KRT5	QT01060325
KRT10	QT00493241
FLG	QT01195929
LOR	QT00248192
SEMA3A	QT00173971
KLK7	QT00150962
KLK5	QT01563177
